# LncRNA interactomes and co-methylation in breast cancer regulation

**DOI:** 10.17305/bb.2025.12333

**Published:** 2025-05-09

**Authors:** Elena A Filippova, Irina V Pronina, Svetlana S Lukina, Alexey M Burdennyy, Tatiana P Kazubskaya, Vitaly I Loginov, Eleonora A Braga

**Affiliations:** 1 Institute of General Pathology and Pathophysiology, Moscow, Russia; 2 N.M. Emanuel Institute of Biochemical Physics, Russian Academy of Science, Moscow, Russia; 3 N.N. Blokhin National Medical Research Center of Oncology, the Ministry of Health of Russia, Moscow, Russia

**Keywords:** LncRNA PCR Array, lncRNAs co-expression, lncRNA genes co-methylation, ADAMTS9-AS2, lncRNA-miRNA-mRNA regulatory axes

## Abstract

Breast cancer is the most commonly diagnosed malignancy in women. Despite advances in diagnostics and treatment, the key molecular mechanisms underlying its development remain incompletely understood. This study aimed to identify novel lncRNA–miRNA–mRNA regulatory networks potentially involved in breast cancer–associated signaling pathways. Using an RT^2^ lncRNA PCR Array and bioinformatic analysis, we identified seven differentially expressed (DE) lncRNAs. Four of these—ADAMTS9-AS2, HAND2-AS1, HOTAIRM1, and MEG3—were prioritized through integrative evaluation. qPCR confirmed their downregulation and aberrant methylation in breast tumor samples. We observed significant positive expression correlations between the pairs ADAMTS9-AS2–MEG3, HAND2-AS1–MEG3, and HOTAIRM1–MEG3, as well as co-methylation among ADAMTS9-AS2–HAND2-AS1, ADAMTS9-AS2–HOTAIRM1, HAND2-AS1–MEG3, and HAND2-AS1–HOTAIRM1, suggesting coordinated regulation. These findings are consistent with data from GEPIA 2.0. Bioinformatic prediction identified TCF7L2 as a common target gene of these lncRNAs, which is involved in the Wnt, Hippo, and MAPK signaling pathways. We also identified several miRNAs interacting with ADAMTS9-AS2. In a cohort of 50 tumor samples, we confirmed inverse associations between ADAMTS9-AS2 expression and levels of miR-106a-5p (*r_s_* ═ –0.46, *P* ═ 0.03) and miR-17-5p (*r_s_* ═ –0.41, *p* ═ 0.04). Collectively, these findings reveal novel co-regulated lncRNA–miRNA axes and suggest their involvement in key signaling networks in breast cancer, providing a foundation for future functional studies and potential therapeutic targeting.

## Introduction

Breast cancer (BC) has long been the most common type of cancer among women [1]. Despite the rapid advancement of modern postgenomic technologies that enable early diagnosis and have led to significant improvements in treatment protocols, the mortality rate remains high. The development of metastases is the leading cause of this high mortality rate in BC patients [2]. Given the remarkable heterogeneity of breast tumors, standard treatment protocols may not be sufficiently effective. Therefore, an in-depth investigation into the mechanisms underlying BC development and progression is essential. Identifying key regulators of signaling pathways involved in BC progression and metastasis could significantly advance the development of effective personalized therapies and improve patient survival rates. Thanks to substantial progress in genome-wide technologies over the past decade, we now have a deeper understanding of the crucial role played by non-coding RNAs (ncRNAs) in regulating genes involved in all stages of tumor development and progression, including relapse and drug resistance [3–5]. Recent research highlights the enormous potential of ncRNAs—specifically their two major classes, long ncRNAs (lncRNAs) and microRNAs (miRNAs)—as diagnostic and prognostic biomarkers, as well as therapeutic targets for BC [6, 7]. lncRNAs are non-protein-coding RNAs approximately 200 nucleotides long that play dynamic regulatory roles in both physiological and pathological processes, including carcinogenesis [5]. miRNAs are evolutionarily conserved, single-stranded RNAs 17–25 nucleotides in length; their 5′-end bases bind to miRNA response elements (MREs), which are approximately seven nucleotides long, in the 3′ untranslated region (3′-UTR) of target mRNAs within the RNA-induced silencing complex (RISC) [8]. lncRNAs and miRNAs regulate gene expression through their interactions within lncRNA–miRNA–mRNA regulatory axes. Therefore, it is crucial to study both the individual regulatory networks of lncRNAs and miRNAs and their points of intersection. The functional significance of these regulatory networks has been established in the development and progression of several cancer types [9]. Depending on their composition, lncRNA–miRNA–mRNA axes can exhibit both tumor-suppressive and oncogenic properties within a specific cancer type. lncRNAs are often considered competing endogenous RNAs (ceRNAs), binding miRNAs in a manner similar to mRNAs and thereby preventing miRNAs from repressing their target mRNAs [10]. Alternative mechanisms of gene regulation by ncRNAs have also been described. For instance, lncRNAs can serve as precursors to miRNAs, which are processed from their exonic or intronic regions. Conversely, miRNAs can regulate lncRNAs by binding to them and promoting their degradation via the RISC complex. Furthermore, lncRNAs and miRNAs can compete for binding to the same mRNA targets, or lncRNAs may mask miRNA binding sites, thereby protecting mRNAs from degradation [11]. Investigating lncRNA–miRNA interactions enables the development of targeted therapies. Identifying and validating novel lncRNA–miRNA–mRNA axes in BC could lead to precise strategies aimed at selectively inhibiting the expression of proteins that drive cancer progression. Given that both lncRNAs and miRNAs can function as tumor suppressors or oncogenes—and that the same molecule may play dual roles even within the same cancer type—targeting the lncRNA–miRNA axis holds promise as an effective therapeutic approach, potentially reducing the risk of off-target effects. This study aimed to identify novel lncRNA–miRNA–mRNA regulatory axes in BC. Additionally, as previously observed in miRNA genes in ovarian cancer [12], co-methylation is often present among hyper-methylated ncRNA genes. Therefore, another objective of this work was to explore cross-regulation of lncRNAs in BC through the methylation of their associated genes.

## Materials and methods

### Bioinformatic analysis

Using the Gene Expression Omnibus (GEO) database, we screened public datasets for differentially expressed (DE) lncRNAs related to “breast cancer,” “gene expression,” and “tissues.” The GSE22820 dataset—comprising samples from 176 primary BC patients and 10 normal breast tissues—was selected for further analysis, as it contained gene expression data from both tumor and adjacent normal tissues. Differential expression analysis was performed using GEO2R, applying a threshold of |logFC| ≥ 1 and *P* < 0.05. To identify overlapping DE genes between the bio-informatic analysis and experimental data obtained using the RT^2^ lncRNA PCR Array (Qiagen) [13], we used the Venn diagram tool available at http://bioinformatics.psb.ugent.be/webtools/Venn/. Epithelial-to-mesenchymal transition (EMT)-associated lncRNAs were filtered from the initial gene set using the GeneCards database (https://www.genecards.org/) with the keyword “EMT.” For further validation, we analyzed expression profiles using the GEPIA 2.0 database (http://gepia.cancer-pku.cn/). Potential miRNAs interacting with the identified lncRNAs were predicted using DIANA-LncBase v3 (https://diana.e-ce.uth.gr/lncbasev3) and RNAInter (http://www.rnainter.org/). Common signaling pathways and biological processes involving these ncRNAs were analyzed using NcPath (http://ncpath.pianlab.cn/) and miRPath v3 (https://dianalab.e-ce.uth.gr/html/mirpathv3/index.php?r=mirpath). To evaluate potential interactions between lncRNAs and mRNAs, the LncRRIsearch web server (http://rtools.cbrc.jp/LncRRIsearch/) was used, while miRNA–mRNA interactions were predicted via the miRWalk 2.0 database (http://mirwalk.umm.uni-heidelberg.de/).

### Collection of samples

A total of 127 paired BC samples (tumor and adjacent histologically normal breast tissue) were collected and morphologically characterized in the Department of Tumor Pathomorphology at the N.N. Blokhin National Medical Research Center of Oncology, in accordance with the WHO classification [14]. Diagnoses were established based on histological findings. The clinical and morphological characteristics of these samples are summarized in Table 1.

**Table 1:** Clinical and pathomorphological parameters of samples from breast cancer patients

**Clinical and histological parameter**	**Number of samples for expression studies** ***N* ═ 50**	**Number of samples for methylation studies** ***N* ═ 127**	
*Stage*	I + II	44	96
	III	6	31
*Tumor size*	T1	14	30
	T2	29	77
	T3	3	10
	T4	4	9
*Lymphnode metastases*	Yes	21	62
	No	29	65
Molecular subtype*	Basal	1	7
	Erb-B2	3	12
	Luminal A	6	16
	Luminal B	37	82
Receptor expression*	ER+/ER−	43/4	94/23
	PR+/PR−	40/7	88/29
	HER2+/HER2−	24/23	62/55
Grade*	G1	8	16
	G2	33	87
	G3	6	22

*Note: Clinical or molecular data were unavailable for some samples; therefore, the total number of cases may vary between parameters. ER: Estrogen receptor; PR: Progesterone receptor; HER2: Human epidermal growth factor receptor 2; G: Grade; Erb-B2: HER2-enriched subtype; Luminal A: Luminal A subtype; Luminal B: Luminal B subtype.

Criteria for inclusion in the study were as follows: (1) a verified diagnosis of BC; (2) a clinical and histological description of the samples obtained (stage, degree of differentiation, tumor size, presence or absence of lymphnode metastases. Exclusion criteria: (1) chemotherapy or radiation therapy; (2) severe chronic infections; (3) severe concomitant somatic pathology (chronic diseases); (4) tumor location other than the breast (after additional histological analysis). The work was conducted in compliance with the principles of voluntariness and confidentiality in accordance with the Declaration of Helsinki of the World Medical Association [15].

To ensure a high tumor cell content (at least 70%), additional histological analysis was performed on microsections (3–5 µm) stained with hematoxylin and eosin. Tissue samples were stored at −70 ^∘^C. Frozen tissues were crushed in liquid nitrogen using the TissueRuptor^®^ II homogenizer (QIAGEN, Hilden, Germany).

### Extraction of total DNA and RNA, assessment of the purity of preparations

High-molecular-weight DNA was isolated using standard phenol-chloroform extraction. DNA concentration was measured based on optical density at 260 nm, and purity was assessed by examining the absorbance spectrum from 230 to 320 nm using a NanoDrop ND-1000 spectrophotometer (Thermo Fisher Scientific, Wilmington, DE, USA). DNA quality was evaluated by electrophoresis on a 0.8% agarose gel using the Sub-Cell GT Horizontal Electrophoresis System (Bio-Rad, Hercules, CA, USA), followed by imaging with the Gel Doc XR+ Gel Documentation System (Bio-Rad). DNA was stored at −20 ^∘^C. Total RNA was extracted from paired breast tissue samples (tumor and adjacent histologically normal tissue) using ExtractRNA #BC032 reagent (Evrogen, Moscow, Russia). RNA samples were treated with RQ1 RNase-Free DNase (Promega, CA, USA) according to the manufacturer’s instructions. RNA concentration was measured at 260 nm using the NanoDrop ND-1000 spectrophotometer (Thermo Fisher Scientific). Purity was further assessed by the 260/280 nm absorbance ratio and by evaluating the shape of the absorbance spectrum from 230 to 320 nm. RNA was stored at −20 ^∘^C.

### Assessment of the differential expression of lncRNAs in clinical samples using the RT^2^ lncRNA PCR Array

Using the RT^2^ lncRNA PCR Array (#LAHS-002Z, QIAGEN Sciences, Frederick, MD, USA), the differential expression of 84 lncRNAs in BC samples was analyzed. Each RT^2^ PCR Array targets genes associated with a specific signaling pathway and includes five housekeeping genes for normalization. Additionally, each array features a panel of proprietary controls to monitor genomic DNA contamination, first-strand synthesis efficiency, and real-time PCR performance. Reverse transcription was performed using the RT^2^ First Strand Kit (QIAGEN Sciences), and qPCR was conducted with the RT^2^ SYBR^®^ Green qPCR Mastermix (Cat. #330529, QIAGEN Sciences). CT values were exported to an Excel file to generate a data table, which was then uploaded to the QIAGEN GeneGlobe Data Analysis Center (http://www.qiagen.com/geneglobe). Samples were designated as control or test groups, and CT values were normalized manually using selected reference genes. The portal calculated fold changes in gene expression using the ΔΔCT method.

### Assessment of the gene methylation and gene expression levels in clinical samples by Real-Time PCR

The methylation levels of lncRNA genes were analyzed via bisulfite DNA conversion followed by quantitative methylation-specific PCR (MS-qPCR) with real-time detection, as described in [16]. Amplification was carried out using the Bio-Rad CFX96 Real-Time PCR Detection System (Bio-Rad, CA, USA) and the qPCRmix-HS SYBR kit (Evrogen, Moscow, Russia), following the manufacturer’s instructions. Oligonucleotide sequences and PCR conditions for the lncRNA genes ADAMTS9-AS2, HAND2-AS1, HOTAIRM1, MEG3, and the control gene ACTB1 are listed in Table 2. Human Genomic DNA (#G1471; Promega, CA, USA) was used as the unmethylated control, while CpG Methylated Human Genomic DNA (#SD1131; Thermo Fisher Scientific, DE, USA) served as the 100% methylation positive control.

**Table 2:** Primer nucleotide sequences and MS-qPCR parameters

**Gene**	Primers for MS-qPCR*, 5′→3′	**T_ann_, ^∘^C**	**PCR product, bp**
*ADAMTS9-AS*	MF: AATTTCGATAGCGTATTTCGGGAGTTAC	59	187
	MR: TCTTAAAATTCCCAAACACATCCTTCCT		
	UF: TTTTGATAGTGTATTTTGGGAGTTATGG	56	238
	UR: AATACTCACCCCCAAACACTAAACTACT		
*HAND2-AS1*	MF: CGAGGTTGGTACGCGGAG	60	121
	MR: CCGACACAACTAAACCGACTC		
	UF: TGGGGTTTTTGTGAGGTTGGTATGT	60	134
	UR: CCCCAACACAACTAAACCAACTCCTC		
*HOTAIRM1*	MF: TTTAGGCGGCGGTAGTTGTTGC	60	212
	MR: ACCCTCTTCCCTTCTCACCTCTCG		
	UF: GATTTGGAGTGTTGGAGTGAAGAAGA	60	219
	UR: TTACAACCACCCAACAAACTCTAACC		
*MEG3*	MF: CGTTAAGTTCGTATTTTTCGATGGATGTT	60	185
	MR: CGCGAATACTTTTTCCCTACGTAAACC		
	UF: TGATGGATGTTTTGAAATTGTTAGGTGTG	60	165
	UR: CAAATACTTTTTCCCTACATAAACCCAACTCA		
ACTB1**	BSF: TGGTGATGGAGGAGGTTTAGTAAGT	60	135
	BSR: AACCAATAAAACCTACTCCTCCCTTAA		

Note: MF/UF is a forward primer for a methylated/unmethylated allele, MR/UR is a reverse primer for a methylated/unmethylated allele. BS-primers for bisulfite-converted DNA. *All oligonucleotides were selected using the SeqBuilder Pro program, which is included in the Lasergene 17.1 software package from DNASTAR, Madison, WI, USA. **Taken from [18]. MS-qPCR: Quantitative methylation-specific PCR.

Reverse transcription of total RNA was performed using the MMLV RT kit #SK021 (Evrogen). Quantitative PCR was conducted on a Bio-Rad CFX96 Real-Time PCR Detection System using the qPCRmix-HS SYBR kit (Evrogen). Primer sequences and PCR conditions for the lncRNAs ADAMTS9-AS2, HOTAIRM1, MEG3, HAND2-AS1, and the reference gene B2M are listed in Table 3. Data were analyzed using relative quantification based on the ΔΔCt method [17]. Changes in lncRNA expression levels of less than two-fold (|ΔΔCt| ≤ 2) were considered not significant. All PCR reactions were performed in three technical replicates.

**Table 3:** Primer nucleotide sequences and RT-qPCR parameters

**Gene**	Primers for RT-qPCR*, 5′→3′	**T_ann_, ^∘^C**	**PCR product, bp**
*ADAMTS9-AS2*	F: CTCCACCCGATCCTTCCATTGA	57.9	199
	R: GGGGGTCTTGCTCTTTCCTTATCC		
*HAND2-AS1*	F: CCCCGAATCTGTAGTGTGGC	59	113
	R: CAGGCGGTGGAGAGGACT		
HOTAIRM1**	F: AGGGGGTTGAAATGTGGGTG	60	162
	R: CTTGAAAGTGGAGAAATAAAGTGCC		
*MEG3*	F: CGGCTGGGTCGGCTGAAGAACT	59	208
	R: CCGTGGCTGTGGAGGGATTT		
B2M***	F: TGACTTTGTCACAGCCCAAGATAG	64	81
	R: CAAATGCGGCATCTTCAAACCTC		

*Note: F: Forward primer; R: Reverse primer; Primers were selected using the SeqBuilder Pro program, which is included in the Lasergene 17.1 software package from DNASTAR, Madison, WI, USA. **Taken from [19], ***Taken from [20].

Reverse transcription of miRNAs was performed using the TaqMan™ MicroRNA Reverse Transcription Kit (Thermo Fisher Scientific) with 6 ng of total RNA per reaction. Quantitative PCR was conducted using the TaqMan Fast Universal PCR Master Mix and TaqMan MicroRNA Assays (Thermo Fisher Scientific) for hsa-miR-106a (Assay ID: 002169) and hsa-miR-17 (Assay ID: 002308), with RNU6B (Assay ID: 001093) and RNU48 (Assay ID: 001006) as reference RNAs. PCR reactions were run on a Bio-Rad CFX96 Real-Time PCR Detection System.

### Ethical statement

The study was conducted in accordance with the Declaration of Helsinki and was approved by the Ethics Committee of the Institute of General Pathology and Pathophysiology (Protocol No. 1, 03/03/2022; Protocol No. 4, 31/08/2023).

### Statistical analysis

The results were analyzed using the R software environment. To evaluate the significance of differences between the studied groups, the non-parametric Mann–Whitney *U* test was applied to independent samples. To assess the impact of promoter CpG island methylation on the regulation of lncRNA gene expression, we calculated Δ β (delta beta) values, representing the difference in methylation levels between tumor and paired normal tissues. Potential co-expression relationships were analyzed using Spearman’s correlation coefficient, with *P* values adjusted using the Benjamini–Hochberg procedure for multiple comparisons. Differences were considered statistically significant at *P* ≤ 0.05.

## Results

### Differential expression of lncRNAs in BC: RT^2^ PCR Array and bioinformatic screening

**Figure 1.**
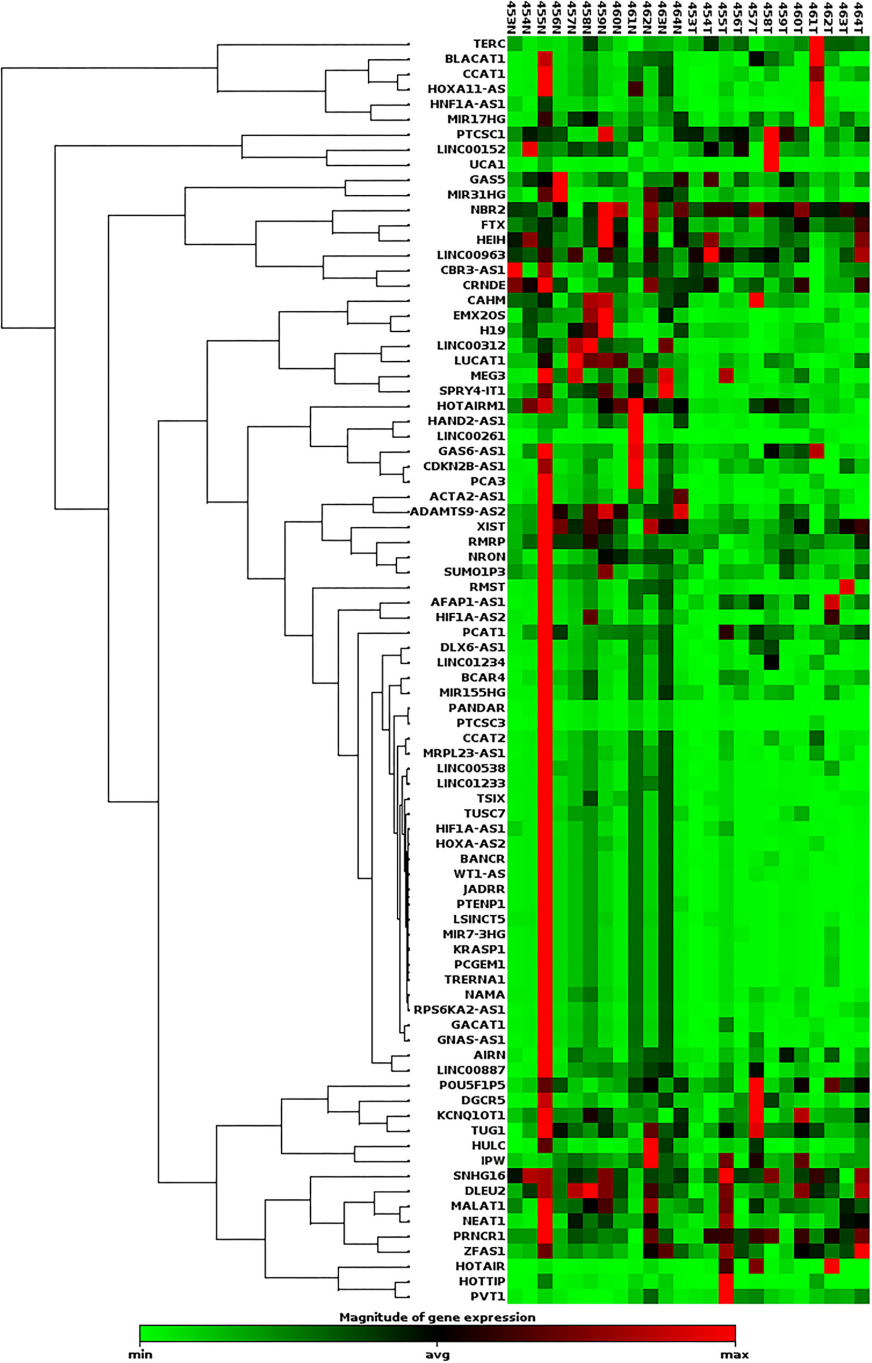
**Differential expression of lncRNAs in 12 paired breast cancer specimens, obtained using the RT^2^ lncRNA PCR Array Gene Expression Analysis kit; lncRNAs with fold change >2 and *P* value <0.05 were considered statistically significant.** LncRNA: Long non-coding RNA.

To comprehensively evaluate the role of lncRNAs in BC, we used the RT^2^ lncRNA PCR Array (QIAGEN Sciences) to assess the expression of 84 lncRNAs in 12 paired tumor and adjacent normal breast tissue samples. The initial screening results, previously reported at the 2nd International Electronic Conference on Biomedicine (2023) [13], revealed a statistically significant decrease in the expression levels of 30 lncRNAs: ACTA2-AS1, ADAMTS9-AS2, BANCR, EMX2OS, H19, HAND2-AS1, HIF1A-AS1, HOTAIRM1, HOXA-AS2, JADRR, KRASP1, LINC00312, LINC00538, LINC01233, LSINCT5, LUCAT1, MEG3, MIR31HG, MIR7-3HG, NAMA, PCA3, PCGEM1, PTENP1, RMRP, SPRY4-IT1, TRERNA1, TSIX, TUSC7, WT1-AS, and XIST. Conversely, the expression levels of two lncRNAs—TERC and RPLP0—were significantly increased (Figure 1; Table S1).

**Figure 2.**
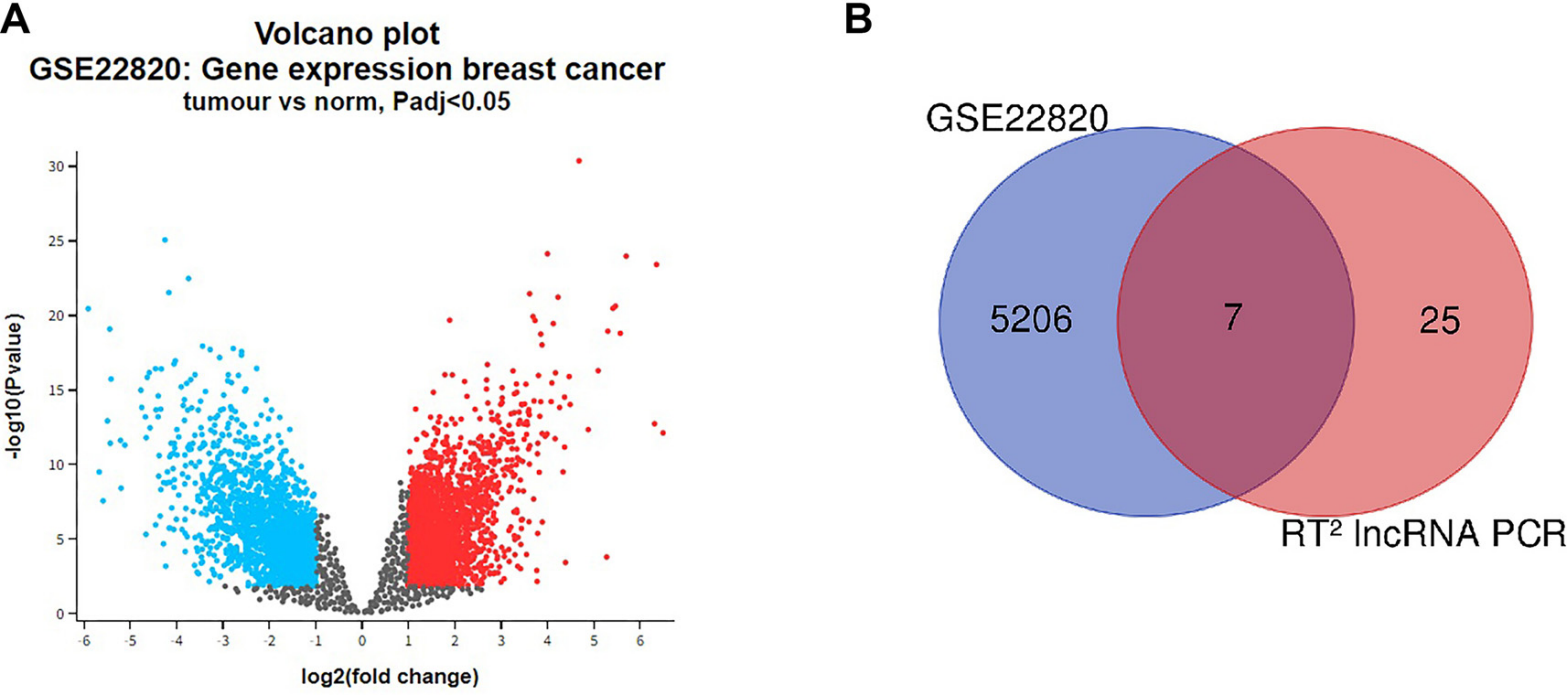
(A) Volcano plot of differentially expressed genes in the GSE22820 data set; (B) Venn diagram of differentially expressed genes in the GSE22820 dataset and those obtained by us experimentally. LncRNA: Long non-coding RNA.

To further underscore the biological significance of our experimental findings, we performed an in silico analysis using the GSE22820 dataset, which includes 176 breast carcinoma and 10 normal tissue samples. This dataset offers robust transcriptional profiling for identifying DE lncRNAs (*P* < 0.05, |log2FC| > 1) relevant to BC. Although clinical annotations are limited, the large sample size and molecular diversity provide valuable insights into tumor heterogeneity and oncogenic pathways. Figure 2A displays a volcano plot of the DE genes. By overlapping lncRNAs identified via GEO2R with our experimental data through Venn diagram analysis, we identified seven commonly dysregulated candidates (HOTAIRM1, HAND2-AS1, ADAMTS9-AS2, EMX2OS, WT1-AS, MIR31HG, and MEG3) (Figure 2B). EMX2OS, WT1-AS, and MIR31HG were subsequently excluded due to non-significant differential expression (*P* > 0.01) between tumor and normal tissues in the TCGA BRCA dataset. This cross-validation step enhanced the robustness of our approach. The remaining four lncRNAs were prioritized for their biological and clinical relevance. Functional enrichment analysis revealed that MEG3 is associated with over 90 EMT- and metastasis-related biological processes, while HOTAIRM1 was linked to three specific metastasis-related GO terms (GO:1904019, GO:1904035, GO:1904036). HAND2-AS1 demonstrated a significant association with overall survival, underscoring its prognostic value. ADAMTS9-AS2 was enriched in the Hippo signaling pathway and several EMT-related processes (e.g., GO:0023061, GO:0002790, GO:0015833), suggesting a potential role in epigenetic regulation and tumor progression.

### Expression and methylation analysis of lncRNAs and their regulatory interplay

**Figure 3.**
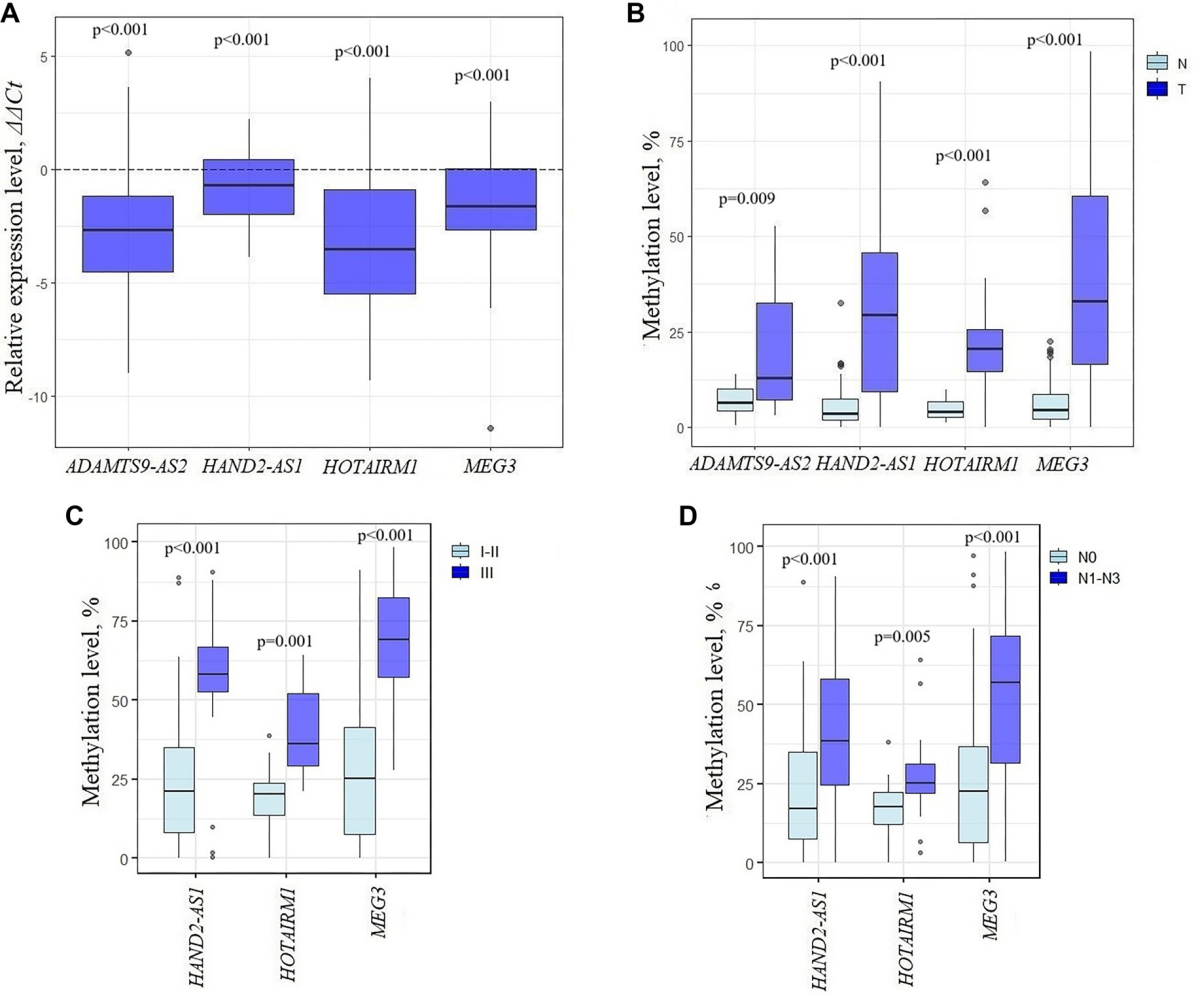
**Expression and promoter methylation of candidate lncRNAs in breast cancer and their clinical relevance.** (A) Expression profile of lncRNAs ADAMTS9-AS2, HOTAIRM1, MEG3, HAND2-AS1 in 50 breast tumor samples; (B) Promoter methylation level of lncRNAs *ADAMTS9-AS2, HOTAIRM1, MEG3*, *HAND2-AS1* in 127 breast tumor samples and in paired normal samples; (C) Association between lncRNA promoter methylation and breast cancer stage (I + II vs III); (D) Association between lncRNA promoter methylation and lymphnode metastases status (N0 vs N1–N3). Statistical significance was assessed using the Mann–Whitney *U* test. LncRNA: Long non-coding RNA.

We next evaluated the expression of four candidate lncRNAs in 50 paired breast tumor and normal tissue samples, confirming significant downregulation of ADAMTS9-AS2, HAND2-AS1, HOTAIRM1, and MEG3 (*P* < 0.001; Figure 3A). Considering promoter hypermethylation as a potential epigenetic mechanism of regulation, we performed MS-qPCR on 127 BC samples. This analysis revealed significant promoter hypermethylation (*P* < 0.05) in all four lncRNAs (Figure 3B), suggesting that hypermethylation may contribute to their transcriptional silencing in BC. Using a subset of 50 paired samples with both methylation and expression data, Spearman’s correlation analysis showed a strong negative trend between promoter methylation (Δ β) and ADAMTS9-AS2 expression (*r_s_* ═ −0.67; *P* ═ 0.07), indicating a possible role for hypermethylation in its silencing. This observation was further supported by integrative analysis using the MethMarkerDB database, which includes TCGA 450K methylation array and RNA-Seq data. These analyses consistently demonstrated a negative correlation between ADAMTS9-AS2 promoter methylation and expression in independent, larger cohorts, reinforcing the role of DNA methylation in regulating ADAMTS9-AS2 expression. Although this trend in the 50-sample cohort was not statistically significant, it appears biologically relevant and merits further validation. Future studies could expand the sample size and employ *in vitro* models with demethylating agents such as 5-azacytidine. Further stratified analysis showed that promoter hypermethylation of HAND2-AS1, HOTAIRM1, and MEG3 was significantly elevated in samples from patients with advanced tumor stage, larger tumor size, and lymph node metastases (Figure 3C and 3D), suggesting a potential link between lncRNA methylation and tumor progression. Notably, MEG3 expression varied significantly across different immunohistochemical subtypes of BC (Kruskal–Wallis test, *P* ═ 0.014), with the most pronounced differences observed between the Erb-B2 and Luminal B subtypes. This finding suggests a possible association between MEG3 dysregulation and specific molecular subtypes. In contrast, no significant associations were observed between the expression of the remaining lncRNAs and molecular subtype, receptor status (ER, PR, HER2), or tumor grade, indicating that their dysregulation may reflect general tumor progression rather than subtype-specific mechanisms.

**Figure 4.**
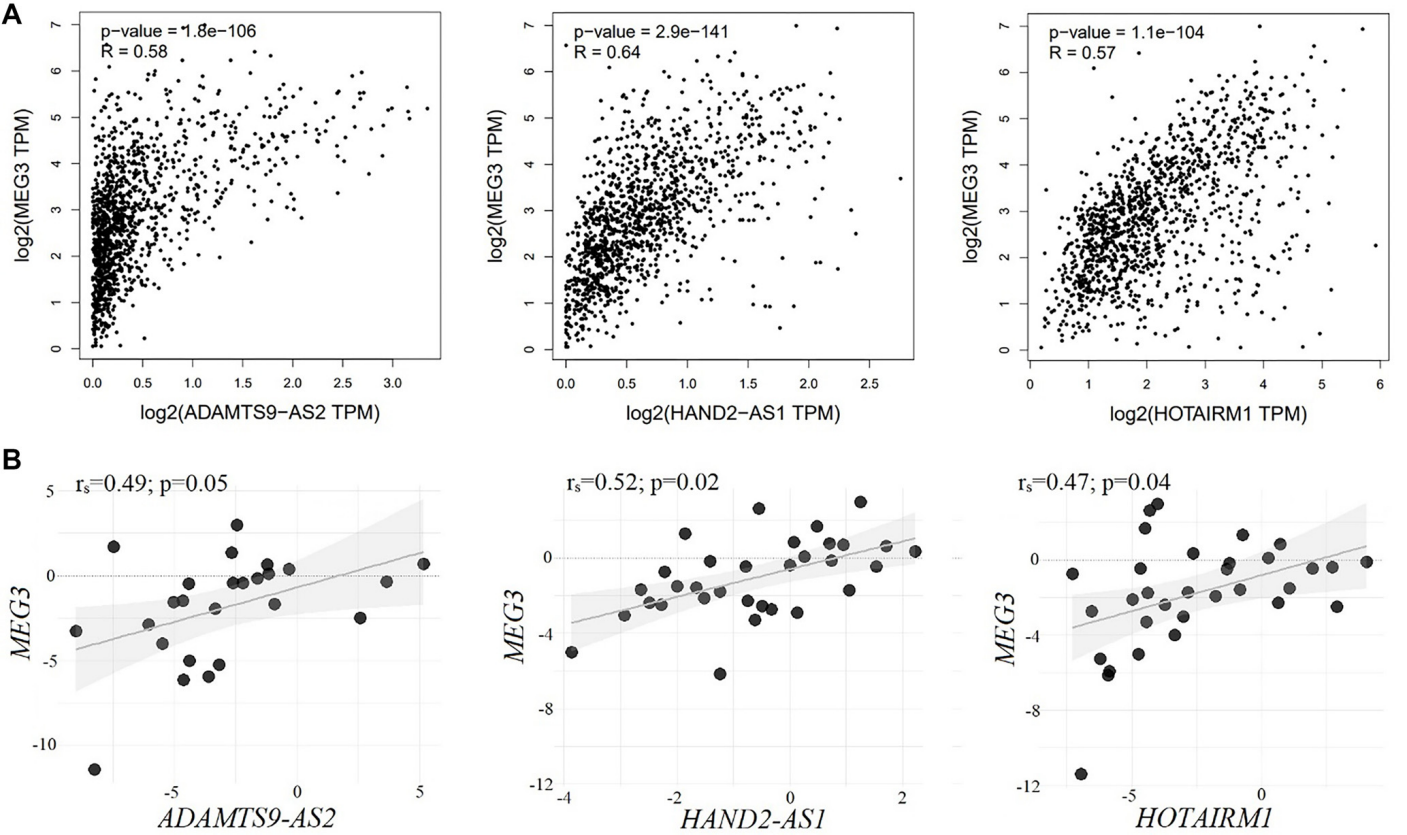
Correlation of relative expression levels of lncRNAs MEG3-ADAMTS9-AS2, MEG3-HAND2-AS1, MEG3-HOTAIRM1: (A) according to TCGA data (built in GEPIA 2.0); (B) according to experimental data; 50 paired breast cancer samples were examined. LncRNA: Long non-coding RNA.

**Table 4:** Spearman’s correlation co-efficient values for co-expressed and co-methylated lncRNA pairs according to data of RT-qPCR in cohort of 50 breast cancer specimens

**lncRNA pairs**	Co-expression* lncRNA (r_s_); ***P* < 0.05**	**Co-expression (GEPIA)** **lncRNA (r_s_);** ***P* < 0.001**	Co-methylation* **lncRNA (rs)** ***P* < 0.001**
MEG3–HOTAIRM1	0.47	0.57	
MEG3–ADAMTS9-AS2	0.49	0.58	
MEG3–HAND2-AS1	0.52	0.64	0.53
HAND2-AS1–ADAMTS9-AS2		0.66	0.70
HAND2-AS1–HOTAIRM1		0.46	0.43
HOTAIRM1–ADAMTS9-AS2		0.49	0.52

*Note: Co-expression/co-methylation experimentally obtained. LncRNA: Long non-coding RNA.

### Pairwise co-expression and co-methylation of lncRNAs in the regulation of common signaling pathways

Our experimental analyses revealed significant co-expression and co-methylation patterns among the lncRNAs MEG3, HOTAIRM1, HAND2-AS1, and ADAMTS9-AS2, suggesting potential coordinated epigenetic regulation (Figure 4). Spearman’s correlation analysis of expression levels showed consistent positive associations between several lncRNA pairs, including MEG3–HOTAIRM1, MEG3–ADAMTS9-AS2, and MEG3–HAND2-AS1. These findings were corroborated by data from the GEPIA 2.0 database, which compiles gene expression profiles from the TCGA BRCA cohort (comprising 1085 tumor and 112 normal breast tissue samples), reinforcing the reproducibility and robustness of our observations (Figure 4, Table 4). In parallel, promoter methylation analysis using MS-qPCR revealed strong positive correlations in methylation levels among the same lncRNA pairs, notably MEG3–HAND2-AS1. Additional co-methylation was observed for ADAMTS9-AS2–HAND2-AS1, HAND2-AS1–HOTAIRM1, and ADAMTS9-AS2–HOTAIRM1, further supporting the hypothesis of shared epigenetic regulation in BC. These co-methylation patterns, consistent with the expression correlations seen in GEPIA, imply potential synergistic regulation of these transcripts (Figure 4, Table 4). Collectively, these findings suggest that the co-expression and co-methylation of these lncRNAs may reflect coordinated involvement in common molecular pathways. To explore the potential biological relevance of these lncRNA interactions, we performed pathway enrichment analysis using the NcPath database, which integrates information from KEGG. This analysis revealed that the co-expressed and co-methylated lncRNAs are associated with seven key cancer-related signaling pathways: Hippo, MAPK, Wnt, adherens junction, focal adhesion, PI3K-Akt, and mTOR. Notably, ADAMTS9-AS2, HOTAIRM1, and MEG3 converge on a shared target—TCF7L2, a transcription factor implicated in the Hippo, Wnt, and adherens junction pathways. Further pathway mapping indicated that these lncRNAs also regulate several extracellular matrix (ECM)-related genes involved in metastasis and cell adhesion. For example, HOTAIRM1 regulates THBS4, MEG3 regulates THBS1, and both ADAMTS9-AS2 and MEG3 share targets, including ERBB4 (MAPK and PI3K-Akt pathways), ITGA3 (PI3K-Akt and focal adhesion), and WNT3 (Hippo, mTOR, and Wnt pathways). Together, these findings suggest that MEG3, HOTAIRM1, HAND2-AS1, and ADAMTS9-AS2 may form a co-regulated epigenetic network involved in the modulation of signaling pathways that drive BC progression.

### Co-expression of miRNAs in common signaling pathways

**Figure 5.**
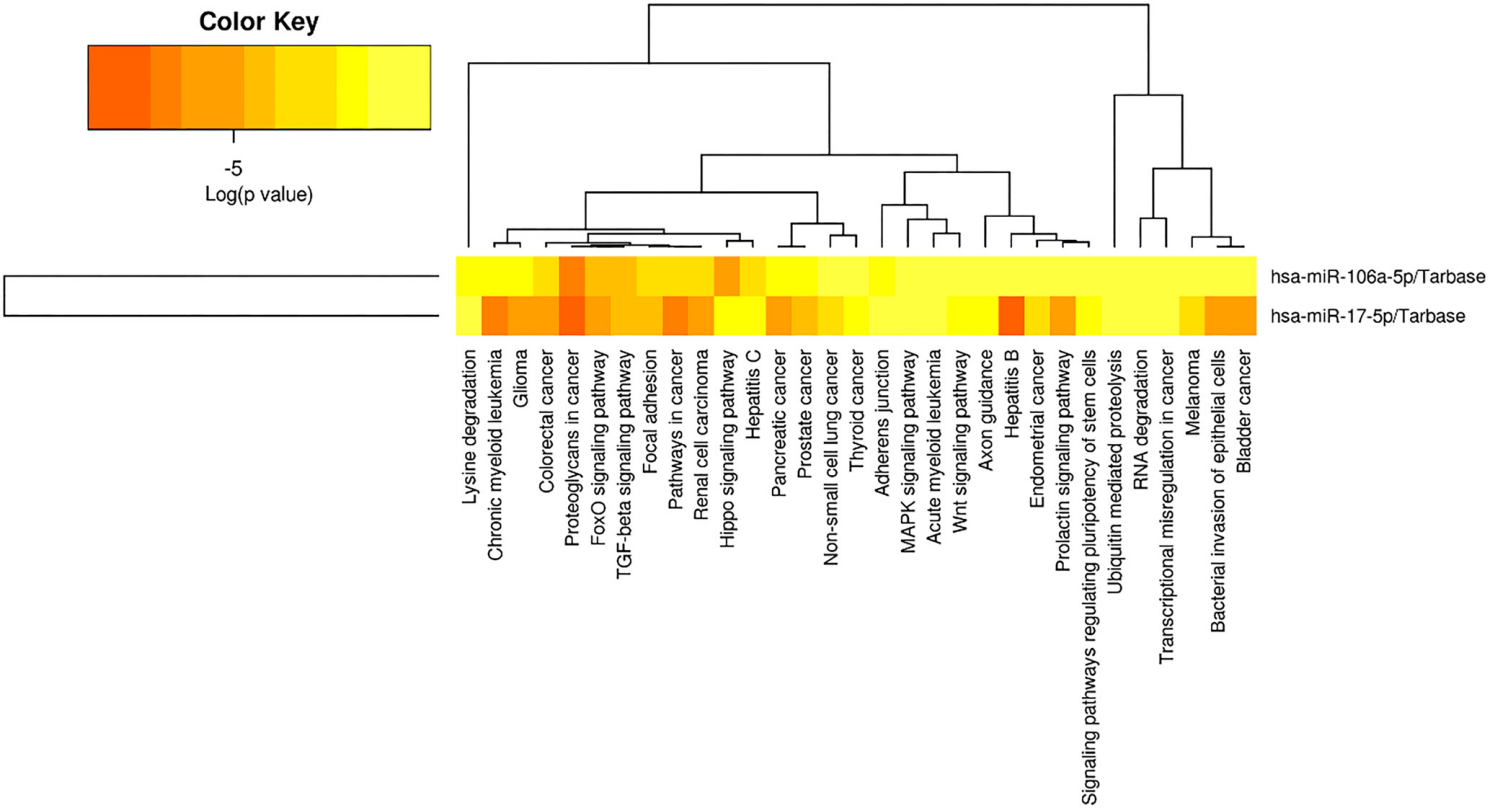
**Heatmap reflecting the signaling pathways in which miRNAs miR-106a-5p and miR-17-5p are involved.** miRNA: MicroRNA.

To investigate the potential regulatory function of lncRNAs mediated by miRNAs, we conducted a bioinformatic analysis to predict miRNAs that might interact with four lncRNAs of interest (ADAMTS9-AS2, HAND2-AS1, HOTAIRM1, and MEG3). Using well-established tools, such as DIANA-LncBase and RNAInter, we identified miR-17-5p and miR-106a-5p as potential interactors. These findings suggest the existence of a functional lncRNA–miRNA regulatory axis that may play a role in BC progression. Analysis of public datasets further highlighted the significant involvement of miR-17-5p and miR-106a-5p in BC, emphasizing their potential contribution to disease progression (Table 5). In our experimental analysis, RT-qPCR performed on 50 paired breast tumor and adjacent normal tissue samples revealed a strong positive correlation between the expression levels of miR-17-5p and miR-106a-5p (*r_s_* ═ 0.78; *P* < 0.001). This high level of co-expression suggests that these miRNAs may function in a coordinated manner and play a significant role in BC development. Additionally, functional enrichment analysis using the miRPath v.3 database identified several key signaling pathways—such as Hippo, MAPK, Wnt, adherens junction, and focal adhesion—as being commonly regulated by both miR-17-5p and miR-106a-5p (Figure 5). Notably, these same pathways were also predicted to be associated with the four lncRNAs based on KEGG data (see previous chapter). The overlap in pathway involvement suggests that these miRNAs and lncRNAs may participate in shared regulatory networks. The observed co-expression patterns from our experimental data support the bioinformatic predictions, reinforcing the hypothesis of common targets, and interconnected signaling pathways.

**Table 5:** Characteristics of miR-17-5p and miR-106a-5p in breast cancer according to bio-informatic databases

**Feature data**	**miR-17-5p**	**miR-106a-5p**	**Source (s)**
Expression level (Tumor vs Normal)	Upregulation (FC ═ 2.38, *P* ═ 1.0e-8)	Upregulation (FC ═ 2.29, *P* ═ 2.7e-8)	TCGA-BRCA
Expression validation	Corroborated (adjusted *P* < 0.05)	GEO: GSE22820	
Clinical correlation	Correlated with stage (*P* ═ 2.28e-2), lymph node involvement (*P* ═ 4.43e-4)	Strongly correlated with distant metastasis (*P* ═ 1.88e-6)	OncoMir, TCGA
Key processes involved	Proliferation, Invasion, Metastasis, EMT, Angiogenesis, Therapy resistance	Literature review	
Major target genes	PTEN, E2F1, ZEB1	Literature review	
Enriched signaling pathways	“Pathways in cancer,” MAPK signaling, Wnt signaling, Adherens junctions, Focal adhesion, Actin cytoskeleton regulation, Cell cycle control	Target gene functional analysis	
Validated target genes (EMT/Metastasis)	VEGFA, TGFBR2, TCF7L2	VEGFA, TGFBR2, TIMP2, HMGA2, TCF7L2	TarBase (validated data)

TCGA-BRCA: The Cancer Genome Atlas - Breast invasive carcinoma; GEO: Gene Expression Omnibus; EMT: Epithelial-to-mesenchymal transition; FC: Fold change; MAPK: Mitogen-activated protein kinase; Wnt: Wingless/integrated signaling pathway; PTEN: Phosphatase and tensin homolog; E2F1: E2F transcription factor 1; ZEB1: Zinc finger E-box binding homeobox 1; VEGFA: Vascular endothelial growth factor A; TGFBR2: Transforming growth factor beta receptor II; TCF7L2: Transcription factor 7 like 2; TIMP2: Tissue inhibitor of metalloproteinases 2; HMGA2: High mobility group AT-hook 2.

### Determining the miRNA-lncRNA “axis” in the regulation of common signaling pathways in BC

**Figure 6.**
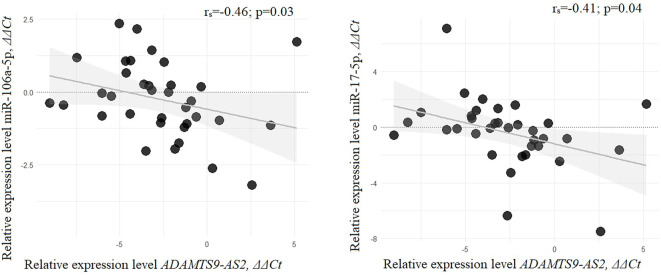
**Correlation of relative expression levels of lncRNA ADAMTS9-AS2 and miRNAs miR-17-5p and miR-106a-5p; 50 paired breast cancer samples were examined.** lncRNA: Long non-coding RNA; miRNA: MicroRNA.

**Figure 7.**
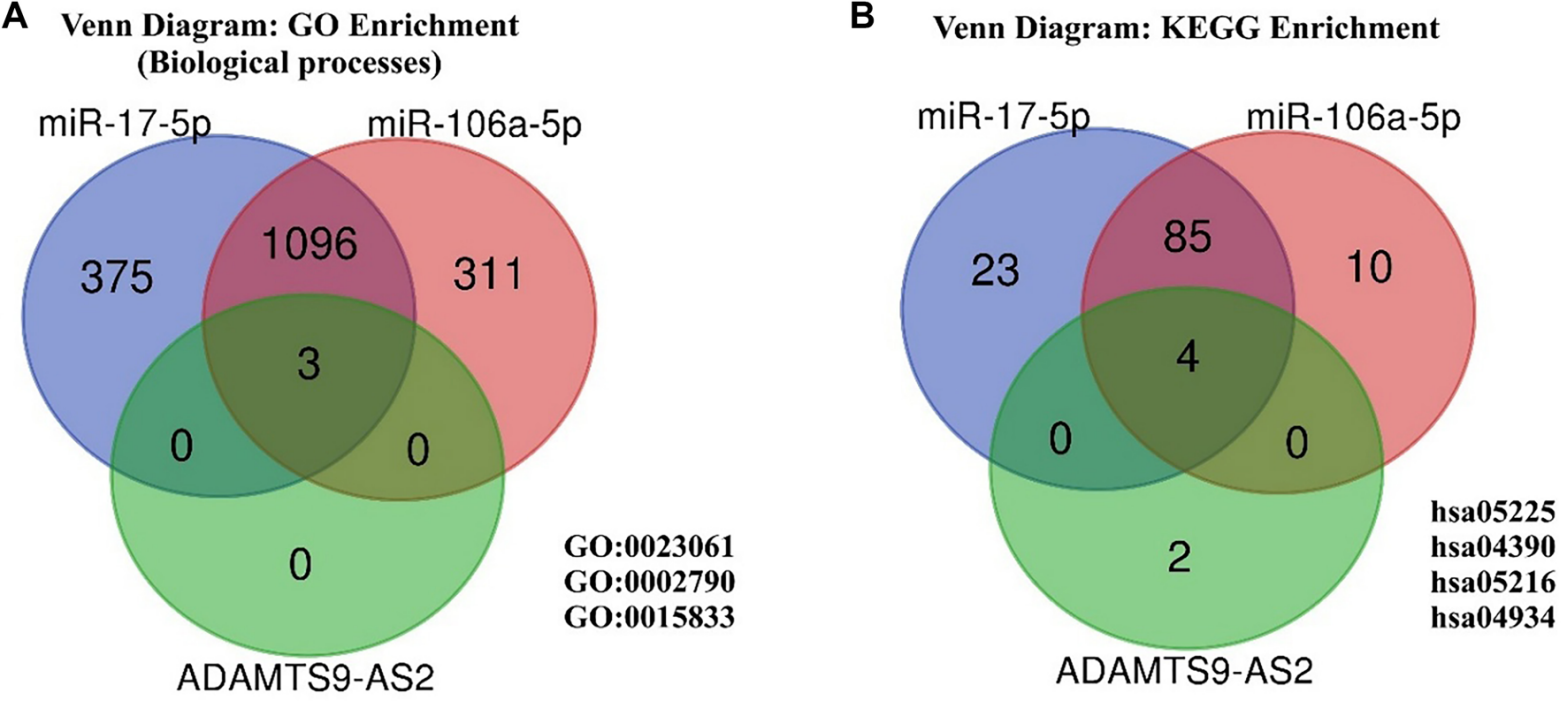
**Intersection of functional enrichment results.** (A) Venn diagram shows the overlapping GO biological processes for miR-17-5p, miR-106a-5p, and ADAMTS9-AS2; (B) Venn diagram shows the overlapping KEGG signaling pathways for miR-17-5p, miR-106a-5p, and ADAMTS9-AS2. GO: Gene Ontology.

**Figure 8.**
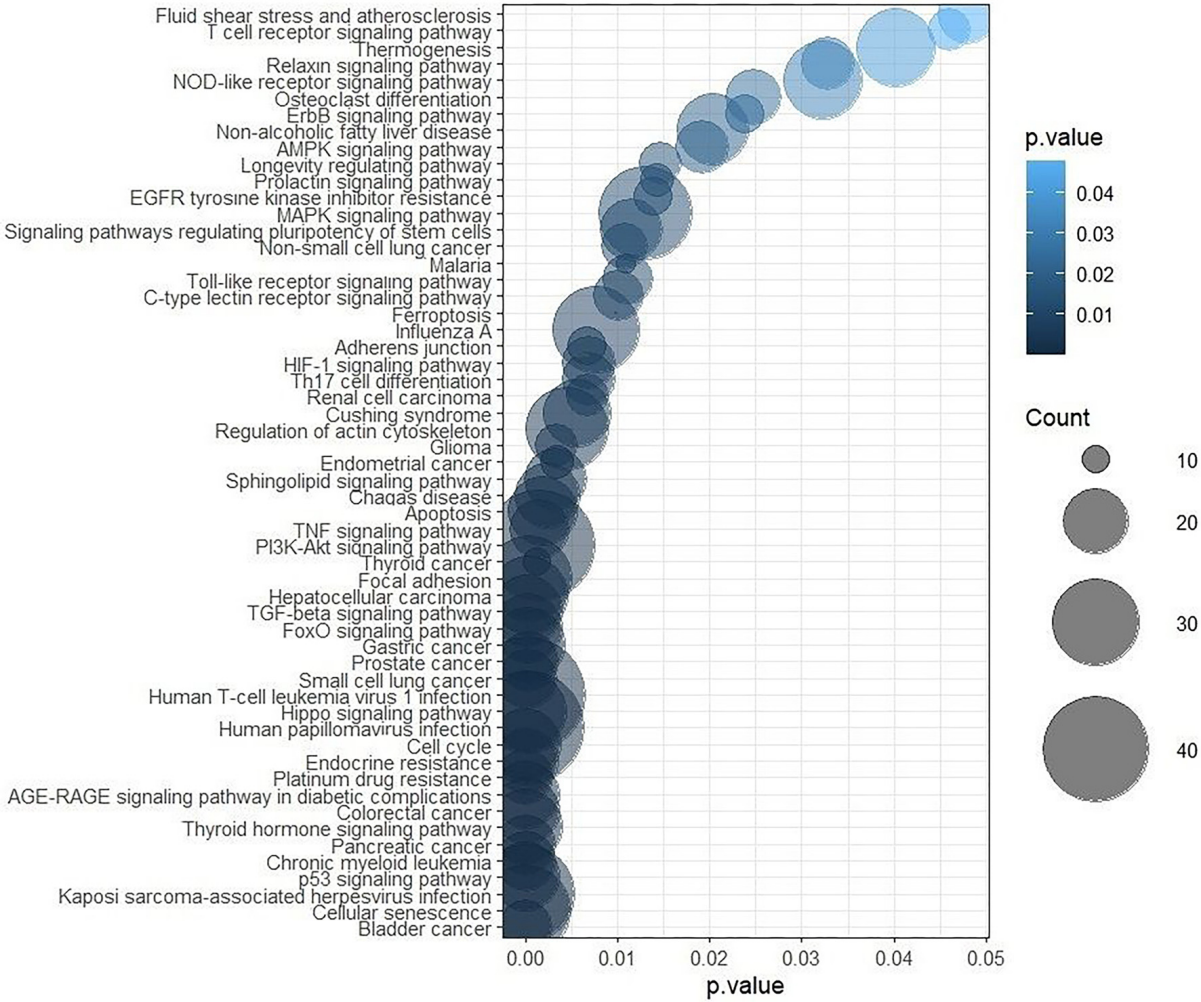
**Results of functional enrichment analysis of the ncPath database involving miRNAs (miR-106a-5p and miR-17-5p) and lncRNA ADAMTS9-AS2.** lncRNA: Long non-coding RNA; miRNA: MicroRNA.

To identify potential miRNA–lncRNA regulatory pairs involved in common signaling pathways, we compared the expression levels of miR-106a-5p and miR-17-5p with those of four lncRNAs using Spearman’s correlation coefficient. A moderate negative correlation was observed between miR-106a-5p and ADAMTS9-AS2 (*r_s_* ═ −0.46, *P* ═ 0.03), as well as between miR-17-5p and ADAMTS9-AS2 (rs ═ −0.41, *P* ═ 0.04) (Figure 6). GO and KEGG enrichment analyses (Figure 7) were performed separately for each miRNA and for ADAMTS9-AS2 to identify shared pathways and functional features. Notably, miR-17-5p was enriched in GO terms directly related to methylation processes (GO:0044027, GO:0141137), highlighting its potential involvement in the regulation of DNA methylation. Additionally, common pathways such as the Hippo signaling pathway—known to play a crucial role in metastasis—suggest that miR-17-5p, miR-106a-5p, and ADAMTS9-AS2 may act in a coordinated manner during BC progression. Further functional enrichment analysis using the ncPath database (Figure 8) also revealed shared signaling pathways involving miR-106a-5p, miR-17-5p, and ADAMTS9-AS2. Moreover, the transcription factor TCF7L2, which is involved in the Wnt and Hippo signaling pathways as well as focal adhesion, may represent a common target of the studied miRNAs and ADAMTS9-AS2. To explore the potential direct interaction between lncRNA ADAMTS9-AS2 and TCF7L2, we analyzed the LncRRIsearch database, which predicted five possible binding sites with a total local base-pairing energy of −68.91 kcal/mol. Similarly, the miRWalk 2.0 database indicated potential direct interactions between TCF7L2 and miR-106a-5p/miR-17-5p, suggesting these miRNAs may bind to the 3′-UTR of TCF7L2. Based on these *in silico* findings, it is plausible that ADAMTS9-AS2 functions as a ceRNA, sequestering miR-106a-5p and miR-17-5p and thereby preventing their interaction with TCF7L2.

**Figure 9.**
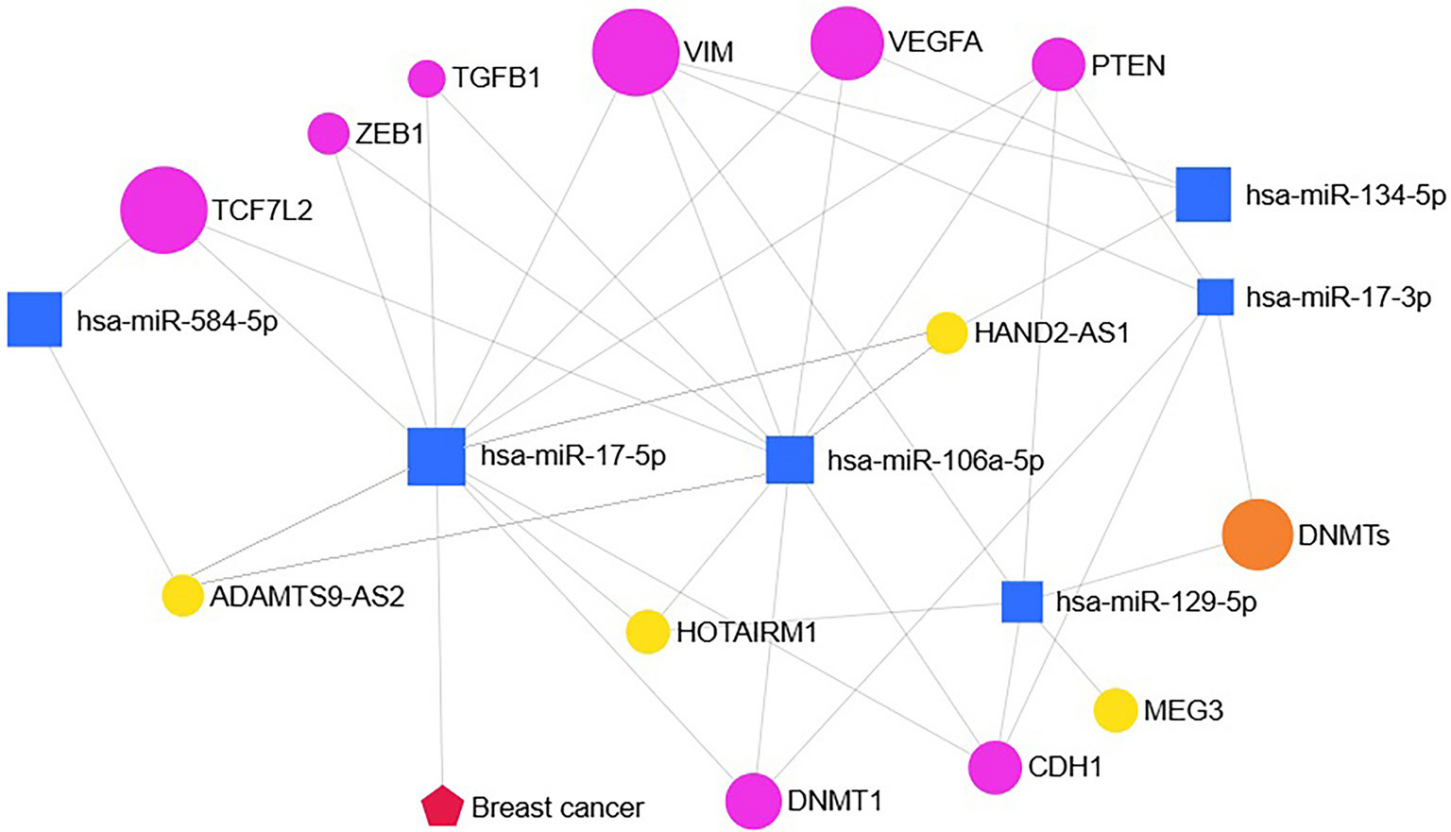
**Regulatory network highlighting potential interactions between lncRNAs, miRNAs, DNMTs, and EMT-related genes in breast cancer.** Yellow circles: LncRNAs; blue squares: MiRNAs; orange circle: DNMTs; magenta circles: EMT/metastasis genes; red pentagon: Breast cancer. Lines indicate potential regulatory relationships. EMT: Epithelial-to-mesenchymal transition; miRNA: MicroRNA; lncRNA: Long non-coding RNA.

A review of the literature in the PubMed database revealed no existing studies on the role of ADAMTS9-AS2 and miR-106a-5p/miR-17-5p in regulating TCF7L2 within the Hippo and Wnt signaling pathways or in relation to cell adhesion in BC, further emphasizing the novelty and relevance of our findings. Although the current study is based on bioinformatic and correlation analyses, future *in vitro* validation—such as dual-luciferase reporter assays, knockdown/overexpression studies, and miRNA mimic/inhibitor experiments—is necessary to confirm the functional significance of the proposed ADAMTS9-AS2/miR-17-5p (or miR-106a-5p)/TCF7L2 axis. Based on bioinformatic predictions, experimental data, and literature review, we constructed a regulatory network illustrating potential interactions among the investigated lncRNAs (ADAMTS9-AS2, HAND2-AS1, HOTAIRM1, MEG3), miRNAs (miR-17-5p, miR-106a-5p, and others), the key DNA methyltransferase DNMT1, and genes associated with EMT and metastasis (including TCF7L2, VIM, VEGFA, ZEB1, CDH1, PTEN, and TGFB1) in the context of BC (Figure 9). Notably, the observed connections between miR-17-5p, miR-106a-5p, and DNMT1 suggest that these miRNAs may influence the epigenetic regulation of DNA methylation, potentially altering the expression of other components of the regulatory network—including lncRNAs and EMT-related genes—and thereby contributing to the complex landscape of tumor progression.

## Discussion

The observed synergy between changes in gene expression and/or methylation levels strongly supports the hypothesis of joint functional roles for the investigated lncRNAs. By integrating robust bioinformatic analyses with experimental validation via RT-qPCR and MS-qPCR, we identified highly significant pairwise co-expression and co-methylation among several lncRNAs involved in key signaling pathways in BC. Notably, we report for the first time specific co-expressed lncRNA pairs—MEG3–HOTAIRM1, MEG3–ADAMTS9-AS2, and MEG3–HAND2-AS1—as well as co-methylated pairs—HAND2-AS1–ADAMTS9-AS2, MEG3–HAND2-AS1, and HOTAIRM1–ADAMTS9-AS2—in BC, with consistent support from the GEPIA 2.0 database. These convergent findings from both experimental data and a large-scale independent dataset reinforce the hypothesis of functional coordination among these lncRNA pairs. The growing body of research on lncRNA interactions in cancer biology underscores the relevance and rapid development of this field. For instance, lncRNAs PSMG3-AS1 and MEG3 negatively regulate each other, affecting proliferation in endometrial carcinoma cells [21], while POU3F3 promotes melanoma cell proliferation by suppressing MEG3 [22]. In glioblastoma, HLA-F-AS1 binds to MEG3, promoting invasion and migration while inhibiting apoptosis [23]. HAND2-AS1 is suppressed by WTAPP1 in non-small cell lung cancer, thereby enhancing invasion and migration [24]. However, to date, no studies have explored interactions among ADAMTS9-AS2, HAND2-AS1, HOTAIRM1, and MEG3 specifically in BC. The positive expression correlations observed here suggest a possible synergistic role in regulating shared signaling pathways, providing new insight into coordinated lncRNA expression patterns in this disease. Functional enrichment analysis using the NcPath database revealed potential involvement of both miRNAs (miR-106a-5p and miR-17-5p) and lncRNAs (ADAMTS9-AS2, HAND2-AS1, HOTAIRM1, MEG3) in signaling pathways associated with BC. This finding aligns with evidence from other tumor types. For example, MEG3 is downregulated in both retinoblastoma and BC cells, and its overexpression suppresses proliferation while inactivating the PI3K/Akt/mTOR pathway, thereby reducing migration and enhancing apoptosis [25, 26]. Similarly, ADAMTS9-AS2 inhibits the PI3K/Akt/mTOR pathway in liver cancer by upregulating ADAMTS9, promoting autophagy, and suppressing migration and invasion [27]. In salivary gland adenoid cystic carcinoma, increased ADAMTS9-AS2 expression lowers ITGA6 levels via miR-143-3p, indirectly affecting both the PI3K/Akt and MEK/Erk pathways [28]. HOTAIRM1 suppresses the PI3K/Akt pathway in gastric cancer by sponging miR-17-5p, leading to increased PTEN expression [29]. HAND2-AS1 similarly inhibits proliferation in non-small-cell lung cancer through modulation of the PI3K/Akt pathway [30].

Based on a literature analysis, we identified MEG3 and HOTAIRM1 as inhibitors of the Wnt/β-catenin pathway in oral squamous cell carcinoma, melanoma, and hepatocellular carcinoma [31–33], suggesting a broader tumor-suppressive role. The convergence of these findings with our bioinformatic predictions supports the potential involvement of these lncRNAs in similar signaling pathways in BC. Although our observations are correlative and may include indirect regulatory effects, they are consistent with previously published data from other cancer types and provide a strong foundation for future functional studies specifically targeting BC. Our assumption regarding the cooperative involvement of the studied lncRNAs in key signaling pathways and biological processes in BC is further supported by observed co-expression and co-methylation patterns, and aligns with existing literature, albeit predominantly from other cancer types. Notably, only one study to date has examined the role of any of the investigated lncRNAs in these pathways specifically in BC [26], highlighting the novelty and potential significance of our broader analysis. In this context, we were the first to report a moderate negative correlation between miR-106a-5p and ADAMTS9-AS2 (*r_s_*= -0.46; *P* ═ 0.03), as well as between miR-17-5p and ADAMTS9-AS2 (*r_s_* ═ −0.41; *P* ═ 0.04), suggesting potential regulatory interactions. Supporting this, the DIANA-LncBase v3 database predicts direct binding between ADAMTS9-AS2 and miR-106a-5p. Moreover, bioinformatic analysis identified TCF7L2—a gene involved in Wnt and Hippo signaling, as well as focal adhesion—as a possible shared downstream target of these miRNAs and ADAMTS9-AS2. While our sample size of 50 may limit the power to detect subtler correlations, we plan to expand the cohort in future studies to enhance the robustness of these findings.

These novel correlations and predicted molecular interactions highlight candidate regulatory axes that may play a role in BC biology and offer a strong conceptual framework for further functional studies aimed at elucidating their mechanistic relevance. Our analysis using LncRRIsearch and miRWalk 2.0 revealed at least five potential binding sites for miR-106a-5p and miR-17-5p within ADAMTS9-AS2 and the 3′-UTR of TCF7L2, supporting the possibility of direct molecular interactions among these components. A PubMed literature search yielded no reports on the involvement of ADAMTS9-AS2 and miR-106a-5p/miR-17-5p in regulating TCF7L2 via the Wnt or Hippo pathways or through cell adhesion in BC, underscoring the originality of our findings and their potential to uncover novel therapeutic targets. A previous study [34] demonstrated that ADAMTS9-AS2 can regulate MEG3 by acting as a molecular sponge for miR-106a-5p in non-invasive bladder cancer. Our findings suggest that a similar regulatory interaction may occur in BC, indicating that the ADAMTS9-AS2–MEG3 axis could represent a conserved mechanism across tumor types. This potential cross-cancer conservation underscores the biological significance of this regulatory pathway and its broader implications in cancer biology. Although direct therapeutic applications remain to be established, our findings provide a valuable foundation for understanding potential regulatory interactions in BC. Further studies are needed to assess the feasibility of targeting these mechanisms using current pharmacological agents or RNA-based therapeutics. Nevertheless, the identified lncRNA–miRNA–mRNA networks represent promising avenues for future translational research and the development of targeted intervention strategies.

## Conclusion

An integrated approach combining bioinformatic analysis with experimental validation revealed coordinated changes in the expression and methylation of four lncRNAs—: MEG3, ADAMTS9-AS2, HAND2-AS1, and HOTAIRM1—as well as the miRNAs miR-106a-5p and miR-17-5p, suggesting potential mutual regulation. Co-expression and co-methylation patterns indicate possible functional cooperation among these molecules, supported by their involvement in shared signaling pathways, including Hippo, MAPK, and Wnt, as well as pathways related to adherens junctions and focal adhesion. Novel interactions were identified between ADAMTS9-AS2 and miR-106a-5p/miR-17-5p, with TCF7L2 predicted as a common downstream target. These findings, based on both experimental data and bioinformatic predictions, support a ceRNA mechanism within the ADAMTS9-AS2–miR-106a-5p/miR-17-5p–TCF7L2 regulatory axis. Computational analysis of binding energies further suggests potential direct interactions between these miRNAs and both ADAMTS9-AS2 and TCF7L2 mRNA, although this requires experimental confirmation. Overall, the identification of novel co-regulated lncRNAs and miRNAs enhances our understanding of regulatory networks in BC and may guide the development of new therapeutic strategies. Characterizing lncRNA–miRNA–mRNA interactions represents a promising avenue for future targeted interventions.

## Supplemental data

**Table S1:** Fold change and *P* value of differentially expressed of lncRNAs obtained using the RT^2^ lncRNA PCR Array Gene Expression Analysis kit in breast cancer

**No**	**Gene**	**FC**	***P* value**		**No**	**Gene**	**FC**	***P* value**
1	ACTA2-AS1	**−4.25**	**0.02**		17	MEG3	**−4.56**	**0.04**
2	ADAMTS9-AS2	**−7.21**	**<0.001**		18	MIR31HG	**−2.87**	**0.04**
3	BANCR	**−4.88**	**0.04**		19	MIR7-3HG	**−5.56**	**0.04**
4	EMX2OS	**−13.2**	**<0.001**		20	NAMA	**−4.85**	**0.04**
5	H19	**−2.94**	**0.02**		21	PCA3	**−5.84**	**0.05**
6	HAND2-AS1	**−6.91**	**0.03**		22	PCGEM1	**−5.94**	**0.04**
7	HIF1A-AS1	**−5.14**	**0.04**		23	PTENP1	**−7.68**	**0.03**
8	HOTAIRM1	**−3.67**	**<0.001**		24	RMRP	**−**1.68	**0.04**
9	HOXA-AS2	**−6.46**	**0.03**		25	SPRY4-IT1	**−5.14**	**<0.001**
10	JADRR	**−6.92**	**0.03**		26	TERC	**2.24**	**0.03**
11	KRASP1	**−6.06**	**0.04**		27	TRERNA1	**−5.8**	**0.04**
12	LINC00312	**−7.83**	**<0.001**		28	TSIX	**−5.79**	**0.02**
13	LINC00538	**−5.85**	**0.04**		29	TUSC7	**−6.37**	**0.03**
14	LINC01233	**−6.62**	**0.02**		30	WT1-AS	**−5.7**	**0.04**
15	LSINCT5	**−5.66**	**0.03**		31	XIST	**−2.34**	**0.04**
16	LUCAT1	**−4.57**	**<0.001**		32	RPLP0	1.85	**<0.001**

The non-bold entries represent mRNAs with changes less than twofold. lncRNA: Long non-coding RNA.

## Data Availability

The data presented in this study are available on reasonable request from the corresponding author.
